# Rare multiple bronchial abnormalities in a patient with congenital heart disease

**DOI:** 10.1002/ccr3.915

**Published:** 2017-03-29

**Authors:** Andrew C. Chatzis, Joanne Sofianidou, Theofili Kousi, Olga Karapanagiotou, Meletios A. Kanakis

**Affiliations:** ^1^ Department of Paediatric and Congenital Cardiac SurgeryOnassis Cardiac Surgery CentreAthensGreece; ^2^ Department of AnaesthesiaOnassis Cardiac Surgery CentreAthensGreece; ^3^ Department of RadiologyOnassis Cardiac Surgery CentreAthensGreece

**Keywords:** Anomaly, bronchus, Congenital Heart Disease, lung

## Abstract

Although many variations regarding lobar or segmental bronchial subdivisions have been described, abnormal bronchi originating from the trachea or main bronchi are relatively rare. These abnormalities can remain undetectable as they usually do not present with symptoms; however, they may pose major obstacles during surgery especially when accompanied by bronchial wall abnormalities.

## Question

What is the appropriate approach to this pathology when combined with congenital heart disease?

## Answer

Meticulous intubation when required to avoid obstruction of the tracheal bronchus and one‐stage repair for the bronchial stenosis is recommended.

## Case History

**Figure 1:**
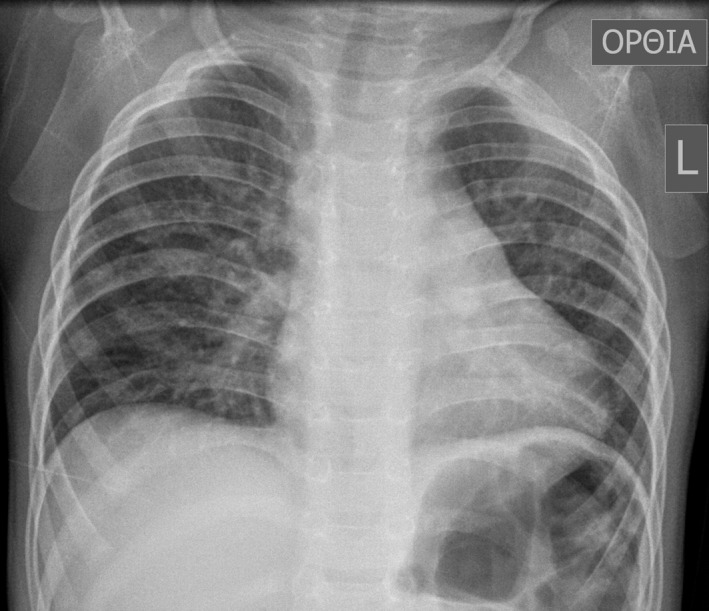
Typical preoperative chest X‐ray of a 16‐month‐old boy with tetralogy of Fallot.

**Figure 2:**
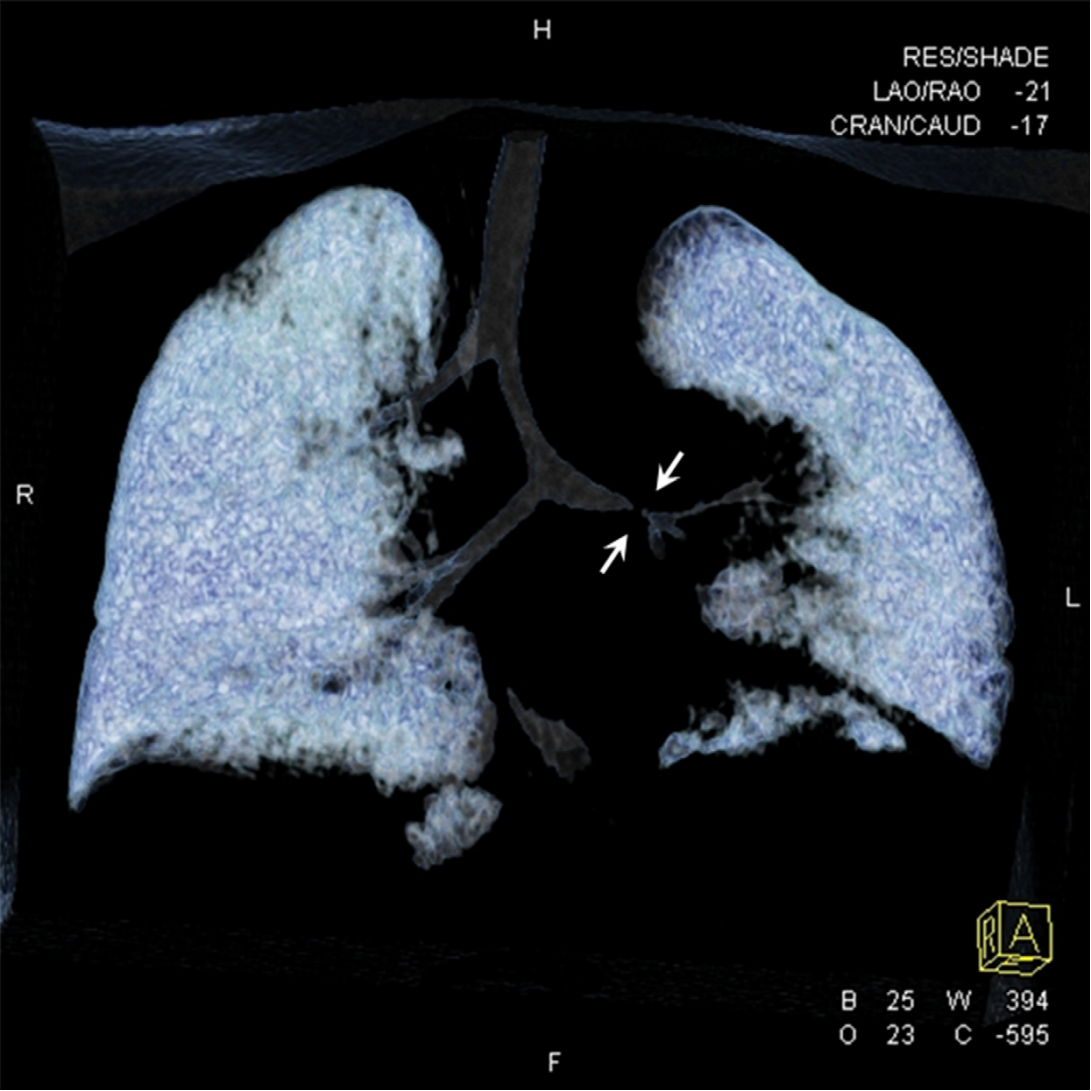
Computed tomography revealed peculiar bronchial anatomy and severe left main bronchial stenosis.

A 16‐month‐old boy with tetralogy of Fallot underwent routine preoperative work‐up for surgical correction (Fig. 1). During the early stage of the procedure mechanical ventilation turned out problematic to manage, the operation was aborted and the patient transferred to ICU for stabilization. Computed tomography revealed peculiar bronchial anatomy and severe left main bronchial stenosis (Fig. 2). These new findings lead to a rescheduled new multidisciplinary surgical plan, that is, combined one‐stage correction for both for the heart defect and the bronchial stenosis.

Plain chest X‐rays are inadequate in displaying bronchial anatomy and computed tomography, especially with appropriate spiral technique on cross‐sectional images, multiplanar and three‐dimensional reconstruction images are required to demonstrate bronchial anomalies 1. Endotracheal intubation of patients with an unknown tracheal bronchus invariably leads to right upper lobe atelectasis 2. Simultaneous repair of associated congenital heart disease and tracheobronchial anomalies although may be accompanied by increased mortality, and morbidity should be the procedure of choice for these patients 3.

## Conflict of Interest

None declared.

## Authorship

ACC: conceived the idea, drafted and revised the manuscript. JS, TK, OK: revised the manuscript for important intellectual content according to their expertise. MAK: collected and analyzed the data. All authors were involved in a major way in the treatment of the patient.

## References

[1] Ghaye, B. , D. Szapiro , J. M. Fanchamps , and R. F. Dondelinger . 2001 Congenital bronchial abnormalities revisited. Radiographics 21:105–119. 11158647 10.1148/radiographics.21.1.g01ja06105

[2] O'Sullivan, B. P. , J. J. Frassica , and S. M. Rayder . 1998 Tracheal bronchus: a cause of prolonged atelectasis in intubated children. Chest 113:537–540. 9498980 10.1378/chest.113.2.537

[3] Xue, B. , B. Liang , S. Wang , L. Zhu , Z. Lu , and Z. Xu . 2015 One‐stage surgical correction of congenital tracheal stenosis complicated with congenital heart disease in infants and young children. J. Card. Surg. 30:97–103. 25109422 10.1111/jocs.12418

